# Supplementary material for: A silicon transporter gene required for healthy growth of rice on land
Source: Nat Commun. 2023 Oct 19;14:6522. doi: 10.1038/s41467-023-42180-y (PMC10587147; doi:10.1038/s41467-023-42180-y)
Supplement: Supplementary file 1 — Supplementary Information [file 41467_2023_42180_MOESM1_ESM.pdf]

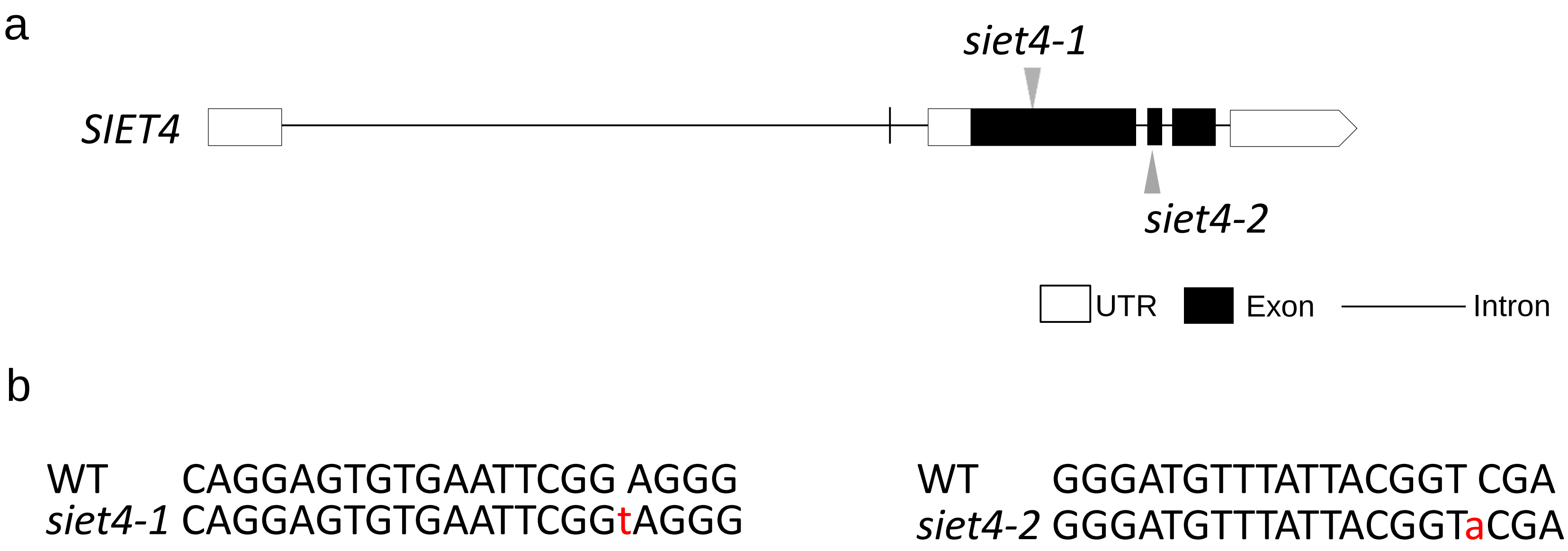

Supplementary Fig. 1. Gene structure of *SIET4* and its mutated sequences in CRISPR/Cas9 mutants. a, Gene structure of *SIET4*. White boxes represent 5' or 3'-UTR regions, black boxes represent exons, the lines between boxes represent introns and arrows show target sites used for generation of knockout lines of *SIET4* with CRISPR/Cas9. **b**, Comparison of sequence between wild-type rice (WT) and two CRISPR/Cas9 lines; *siet4-1* with 1-bp insertion (red color) at the first exon and *siet4-2* with 1-bp insertion at the second exon..

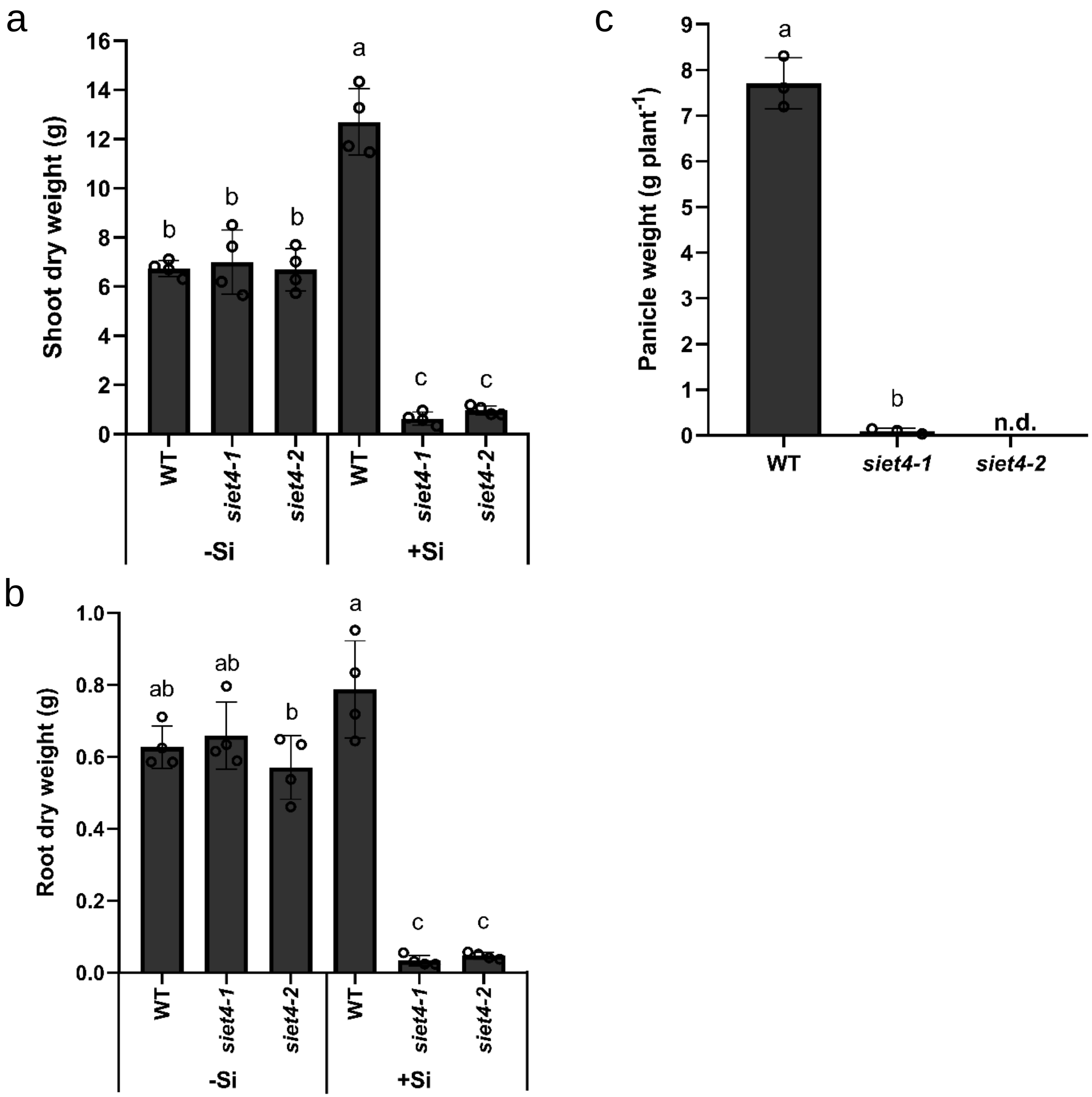

Supplementary Fig. 2. Comparison of the dry weight of shoot, root and grain between wild-type rice (WT) and *siet4* mutants. a-b, Dry weight of shoot (**a**) and root (**b**). Both WT and *siet4* mutants were grown in a nutrient solution containing 0 (-Si) or 1 mM Si (+Si) until maturation. **c**, Panicle weight. Both WT and *siet4* mutants were grown in pot soil until maturation and the panicle weight was recorded after drying. n.d., not-detected. Data are means ± SD (n=4 biological independent plants for **a** and **b**, n=3 biologically independent plants for **c**). Different letters indicate significant difference compared with the WT (P<0.05).

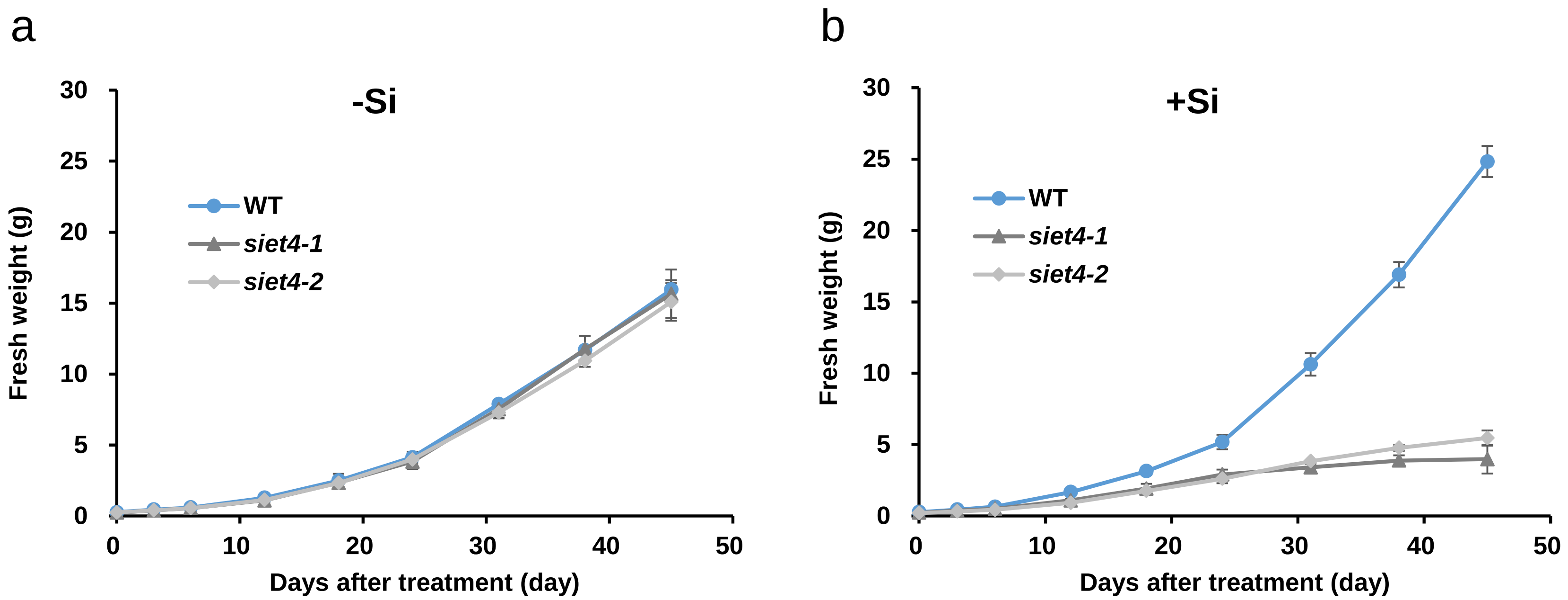

Supplementary Fig. 3. Time-dependent growth curve. The wild-type rice (WT) and *siet4* mutants were grown in a nutrient solution free of Si (**a**, -Si) or with 1 mM Si (**b**, +Si). At time points indicated, the fresh weight was recorded. Data are means ± SD (n=4-10 biologically independent plants).

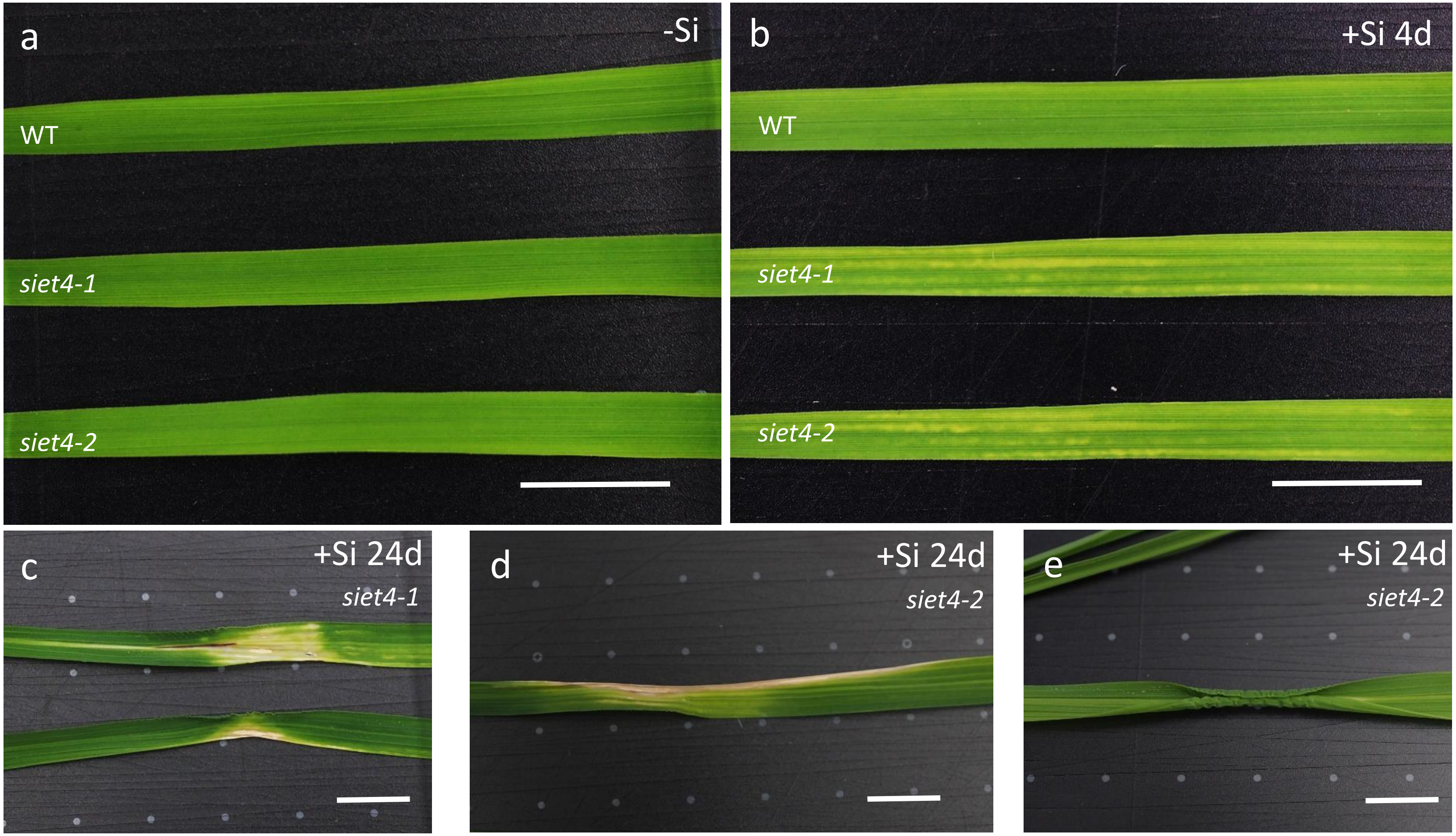

Supplementary Fig. 4. Symptoms observed in leaves of *siet4* mutants. The wild-type rice (WT) and *siet4* mutants were grown in a nutrient solution free of Si (**a**, -Si) or with 1 mM Si (**b-e**, +Si). On day 4 (**a-b**) and day 24 (**c-e**), the symptoms were photographed. Bar = 1 cm

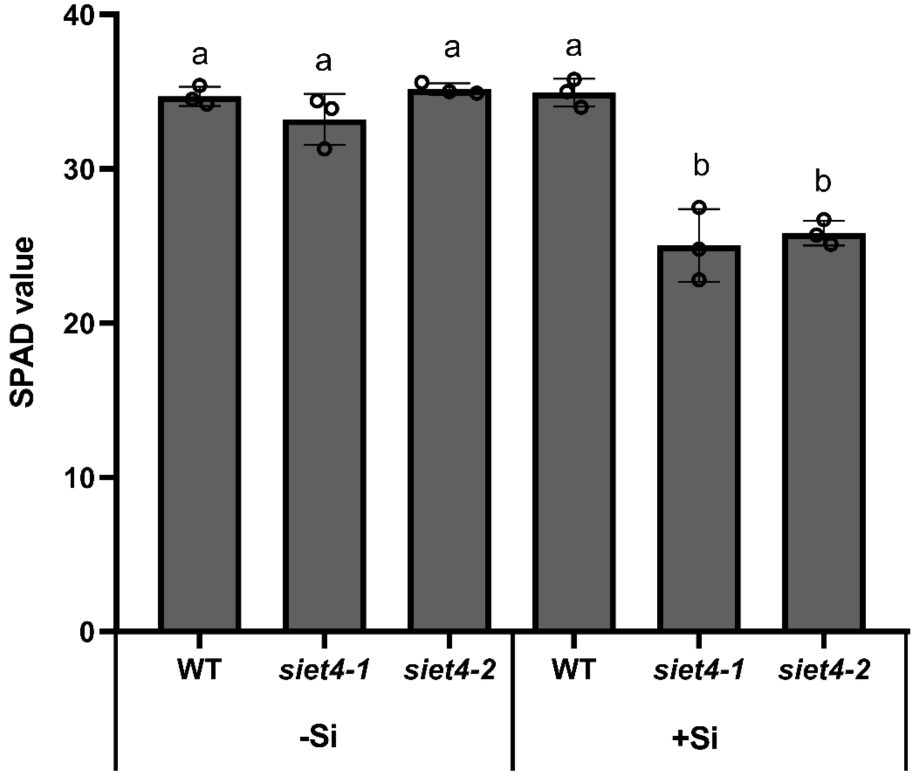

Supplementary Fig. 5. Comparison of SPAD value between wild-type rice (WT) and two *siet4* mutants. Seedlings (20-d-old) were grown in a nutrient solution free of Si (-Si) or with 1 mM Si (+Si). After 5 days, the SPAD value was measured on the youngest fully expanded leaf. Data are means ± SD (n=4 biologically independent plants). Statistical analysis was performed by ANOVA followed by Tukey's test. Different letters indicate significant differences (P<0.05).

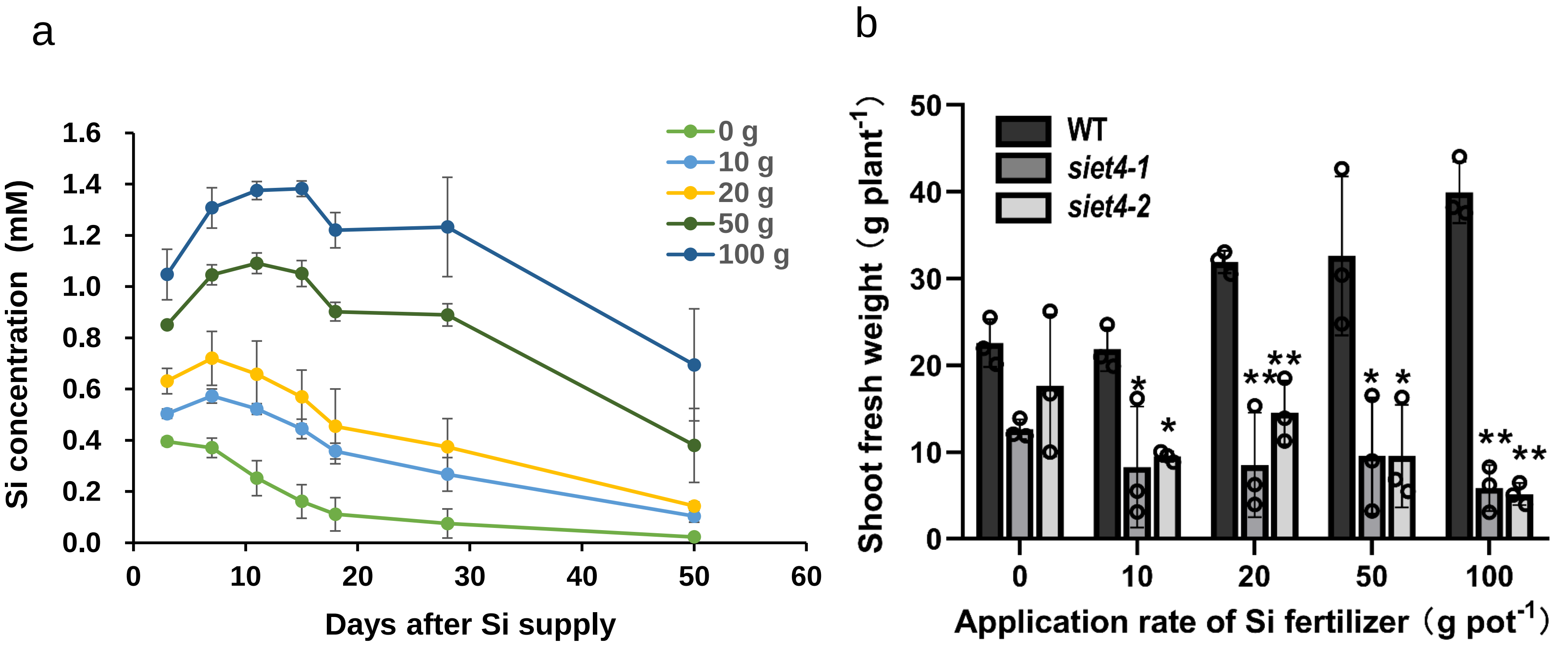

Supplementary Fig. 6. Effect of different Si application rates on the growth of *siet4*. (a) Si concentration in solution collected from pots with different water silica application. (**b**) Shoot fresh weight of wild-type rice (WT) and two *siet4* mutants grown with different water silica application. Seedlings (27-d-old) of both the wild-type rice (WT) and *siet4* mutants were transferred to a pot together filled with river sand with three independent replicates. Water silica was added to each pot at different rate from 0 to 100 g. The plants were supplied with Kimura B nutrient solution and grown for 50 days. Data are means ± SD (n=3 biologically independent plants). Statistical analysis was performed by ANOVA followed by Tukey's test. * and ** indicate significant differences compared with the WT in each treatment (P<0.05 or <0.01).

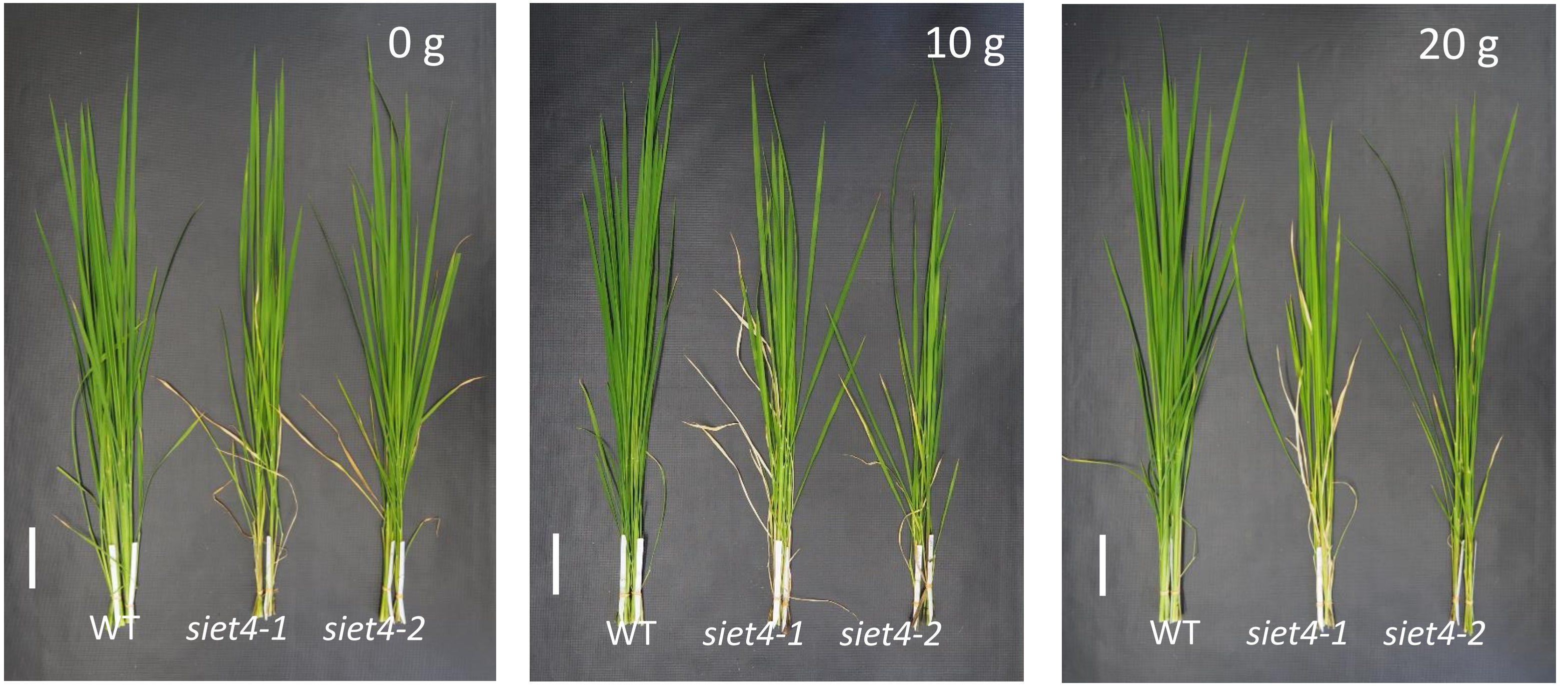

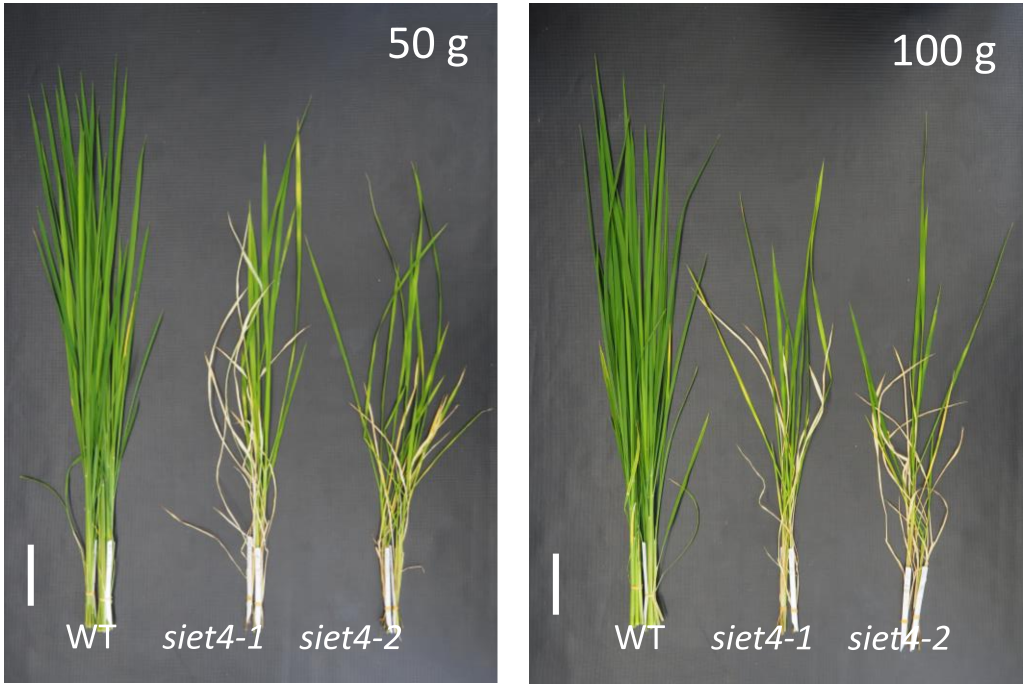

Supplementary Fig. 7. Phenotype of wild-type rice (WT) and *siet4* mutants grown with different Si applications. Seedlings (27-d-old) of both the wild-type rice (WT) and *siet4* mutants were transferred to a pot filled with river sand. Water silica was added to each pot at different rates from 0 to 100 g. After a 50-day growth, the plants were photographed. Bar = 10 cm.

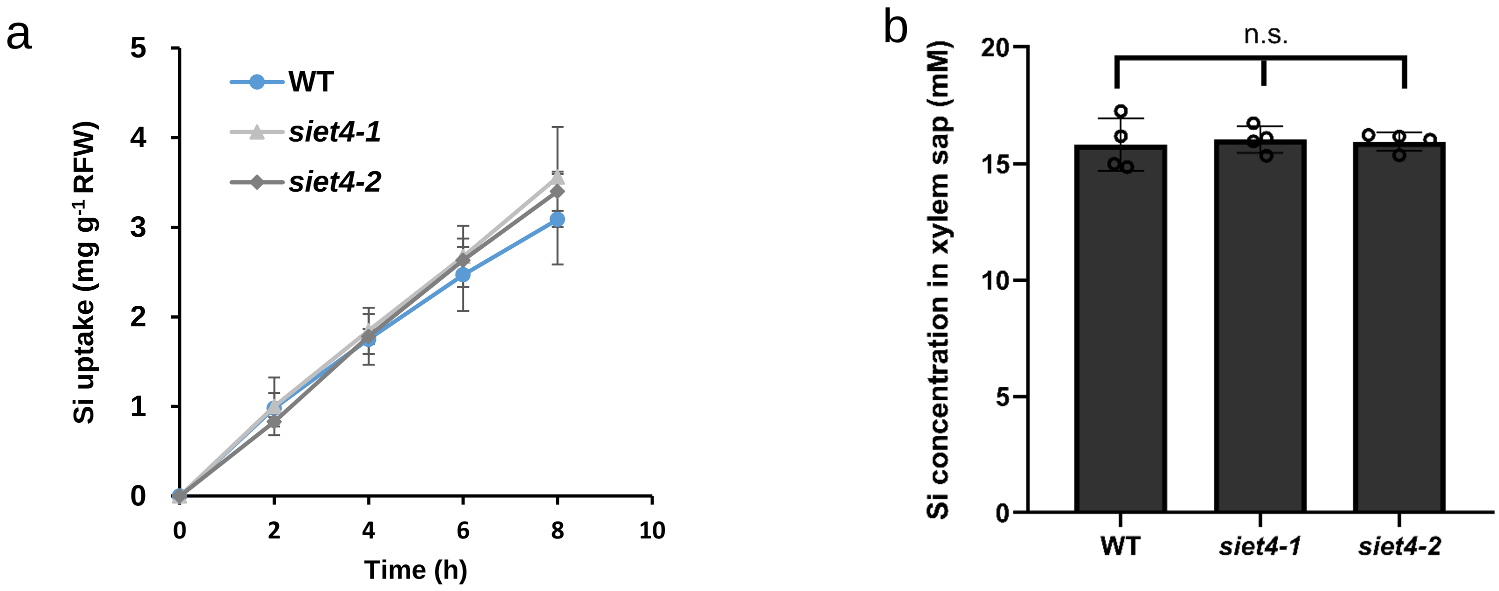

Supplementary Fig. 8. Time-dependent Si uptake and Si concentration in xylem sap. a, Short-term Si uptake. Seedlings (26-d-old) of the wild-type rice (WT) and two *siet4* mutants were exposed to a solution containing 0.5 mM Si. At different time points indicated, Si uptake was determined. Data are means ± SD (n=4). **b**, Si concentration in xylem sap. Seedlings (30-d-old) of two *siet4* mutants and their WT were transferred to a nutrient solution containing 1 mM Si. After 2 h, xylem sap was collected. Data are means ± SD (n=4 biologically independent plants). Statistical analysis was performed by ANOVA followed by Tukey's test. n.s., not significant (P>0.05). RFW, root fresh weight.

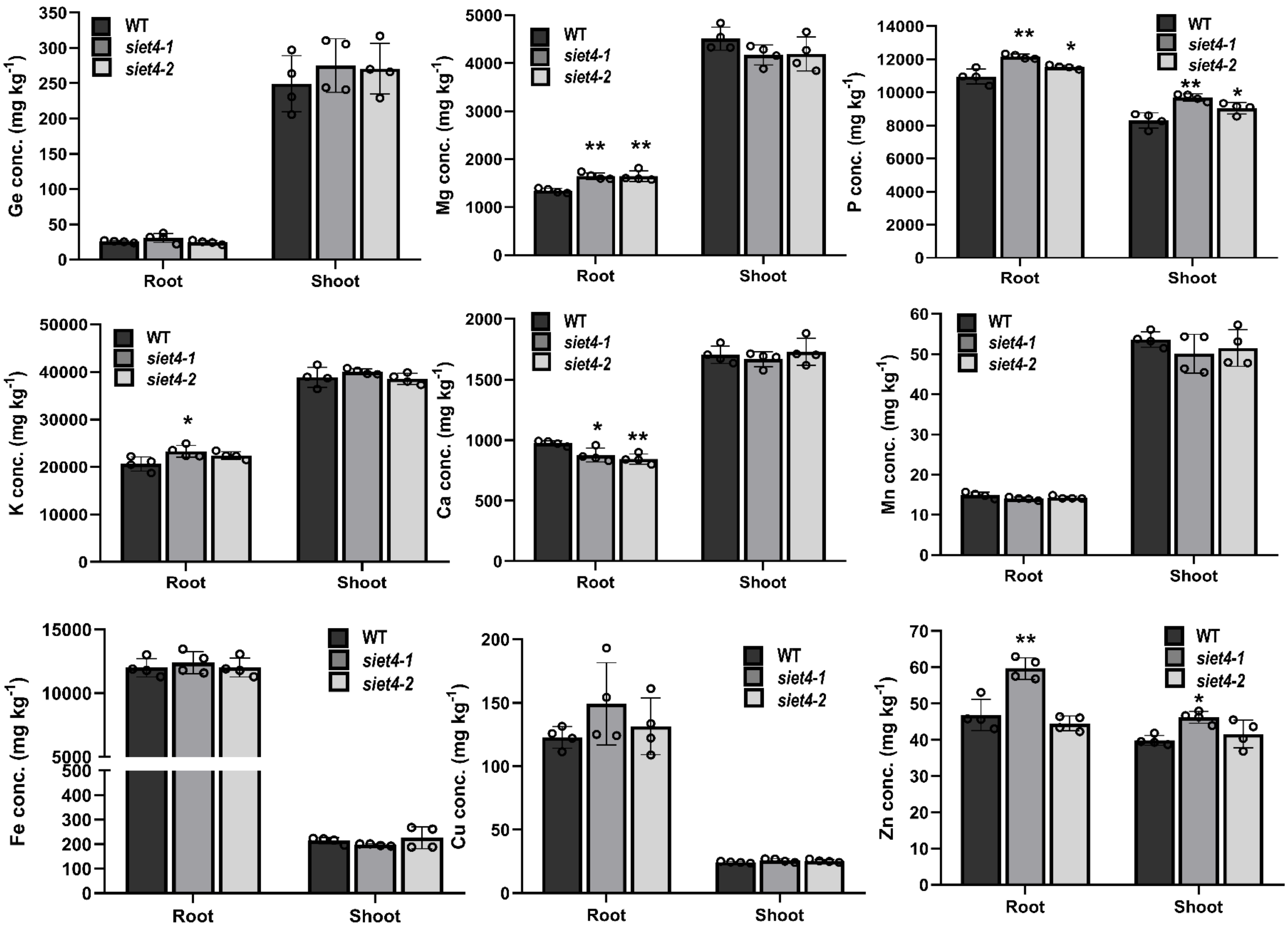

Supplementary Fig. 9. Comparison of mineral concentration in roots and shoots between the wild-type rice (WT) and *siet4* mutants. Seedlings (31-d-old) grown in a nutrient solution free of Si were exposed to a nutrient solution containing 5 µM Ge. After 24 h, the roots and shoots were separately harvested and subjected to determination of mineral elements by ICP-MS. Data are means ± SD (n=4 biologically independent plants). Statistical analysis was performed by ANOVA followed by Tukey's test. * and ** indicate significant difference compared with the WT (*P*<0.05 or <0.01).

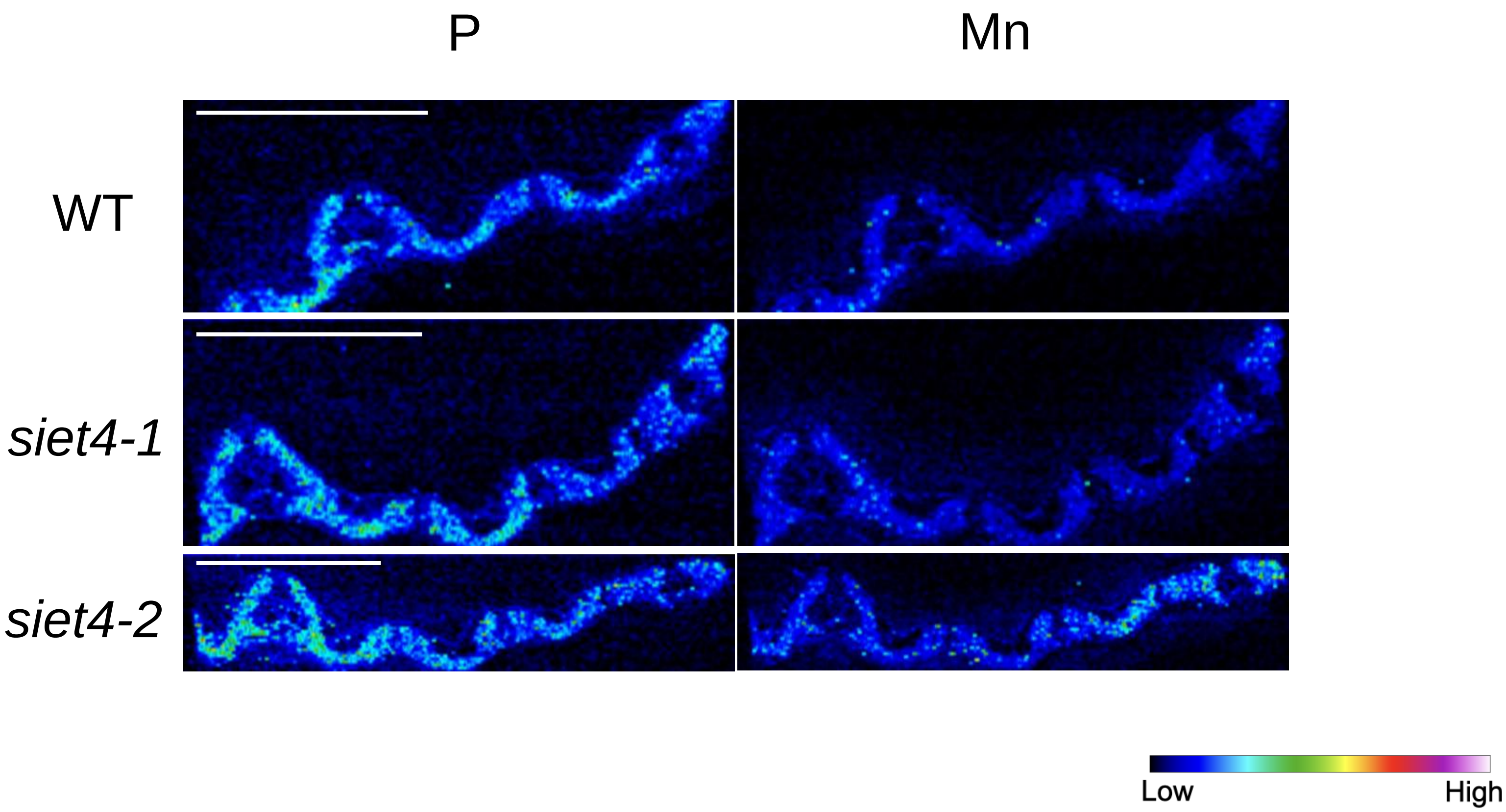

Supplementary Fig. 10. Deposition pattern of phosphorus (P) and manganese (Mn) in rice leaf blade. Seedlings (27-d-old) of the wild-type rice (WT) and two *siet4* mutants (*siet4-1* and *siet4-2*) were exposed to a nutrient solution containing 1 mM Si. After 6 d, leaf blade of the youngest fully expanded leaf was sampled for detection by LA-ICP-MS analysis. Bar = 300 μm.

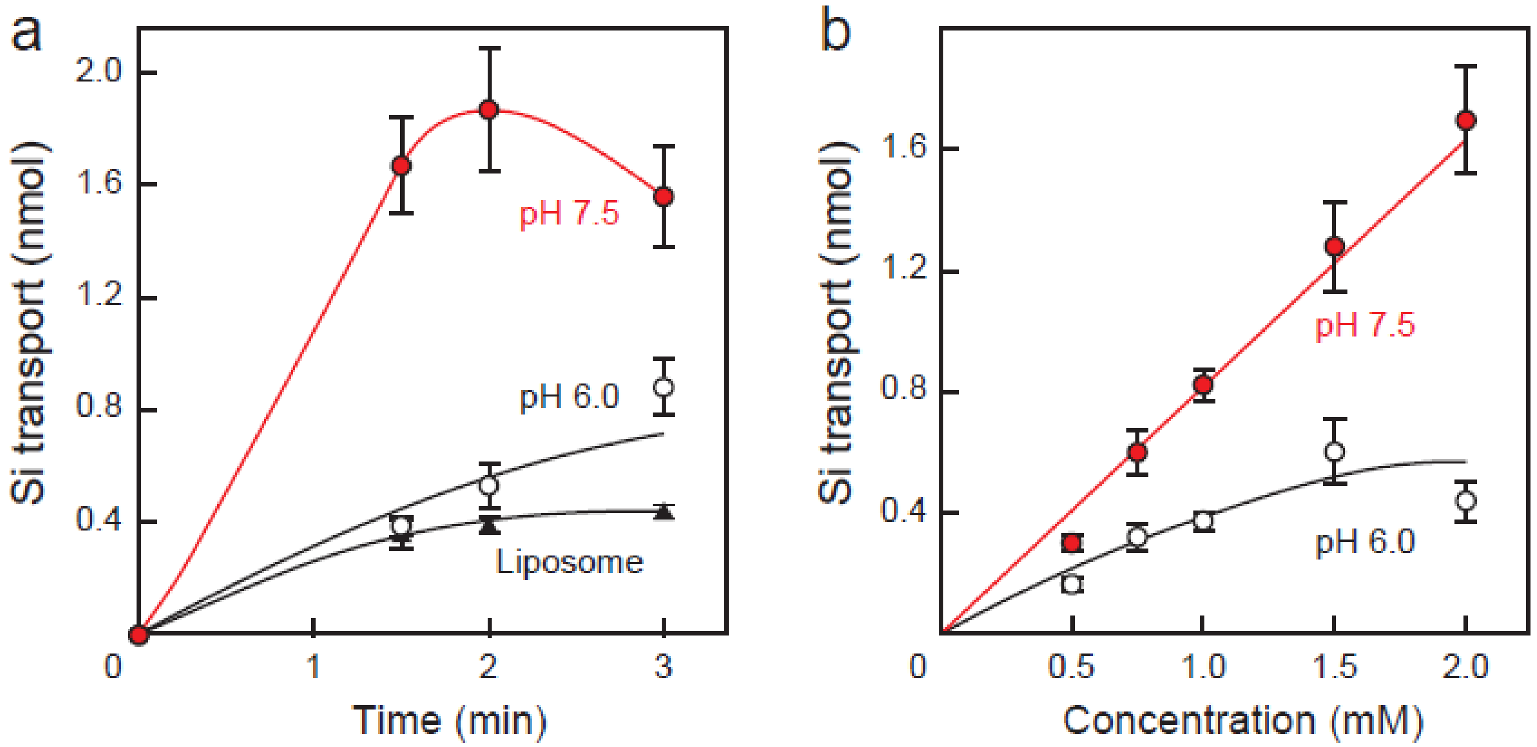

Supplementary Fig. 11. Characterization of SIET4-mediated transport for Si. a, Time-course of SIET4-mediated Si transport. The proteoliposomes with or without SIET4 at pH 6.0 inside were incubated in a buffer solution containing 1 mM Si at either pH 6.0 or pH 7.5. At different time point indicated, the proteoliposomes were sampled and subjected to determination of Si by ICP-MS. **b**, Dose-dependence of SIET4-mediated Si transport. The proteoliposomes with SIET4 at pH 6.0 inside were incubated in a buffer solution containing different Si concentrations at either pH 6.0 or pH 7.5. After 1.5 min incubation, the proteoliposomes were sampled and subjected to determination of Si by ICP-MS. Data are mean ± s.e.m (n=6 independent experiments).

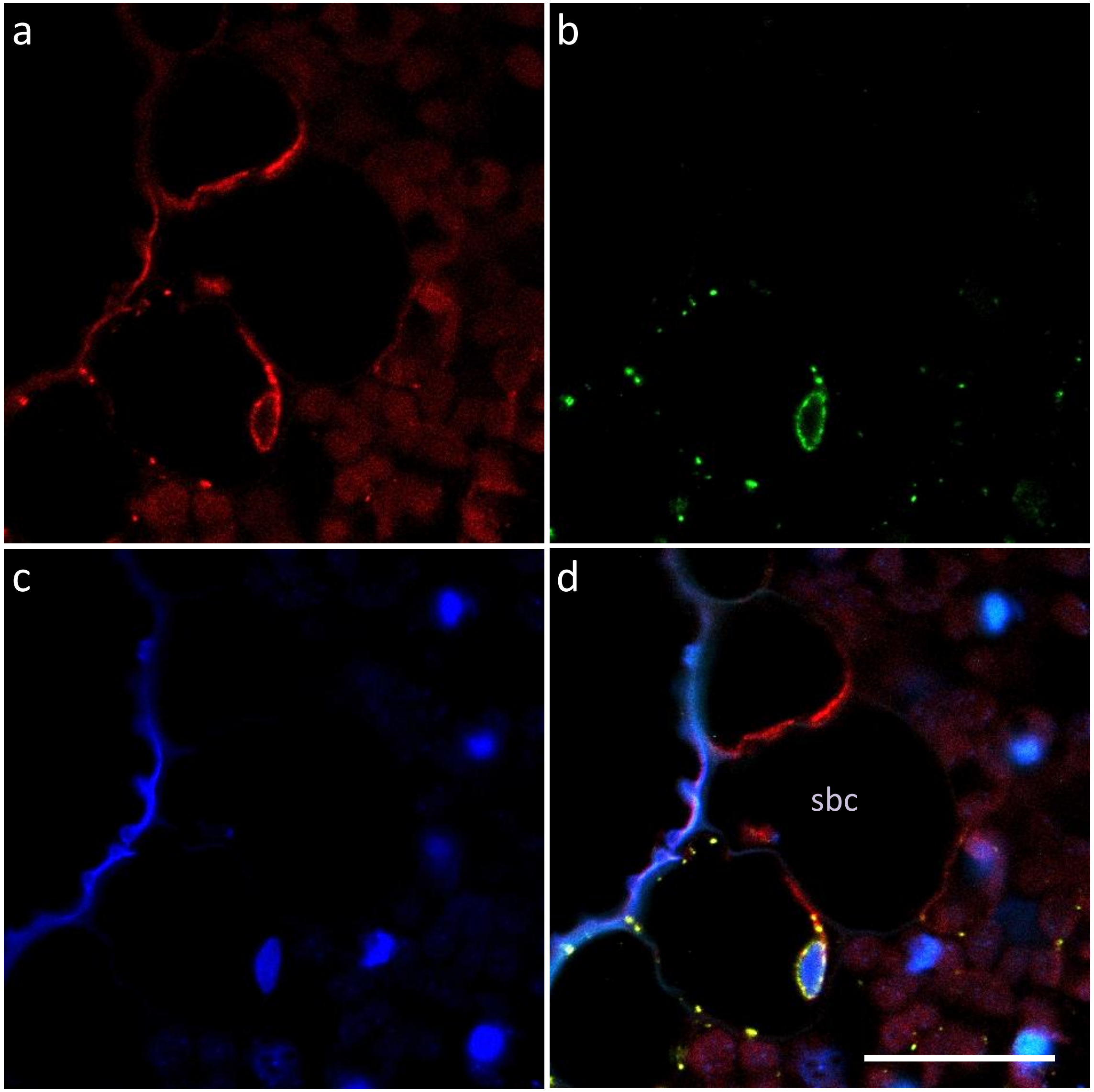

Supplementary Fig. 12: Sub-cellular localization of SIET4. Double immunostaining with SIET4 and HDEL (ER marker) antibodies was performed in mature leaf blade of the wild-type rice exposed to 1 mM Si for 1 d. **a**, Signal from SIET4 (red). **b**, Signal from ER (green). **c**, Signal from nuclei stained by DAPI and epidermal cell wall autofluorescence (blue). **d**, Overlay image of (**a-c**). sbc, silicified bulliform cell. Bar = 20 µm.

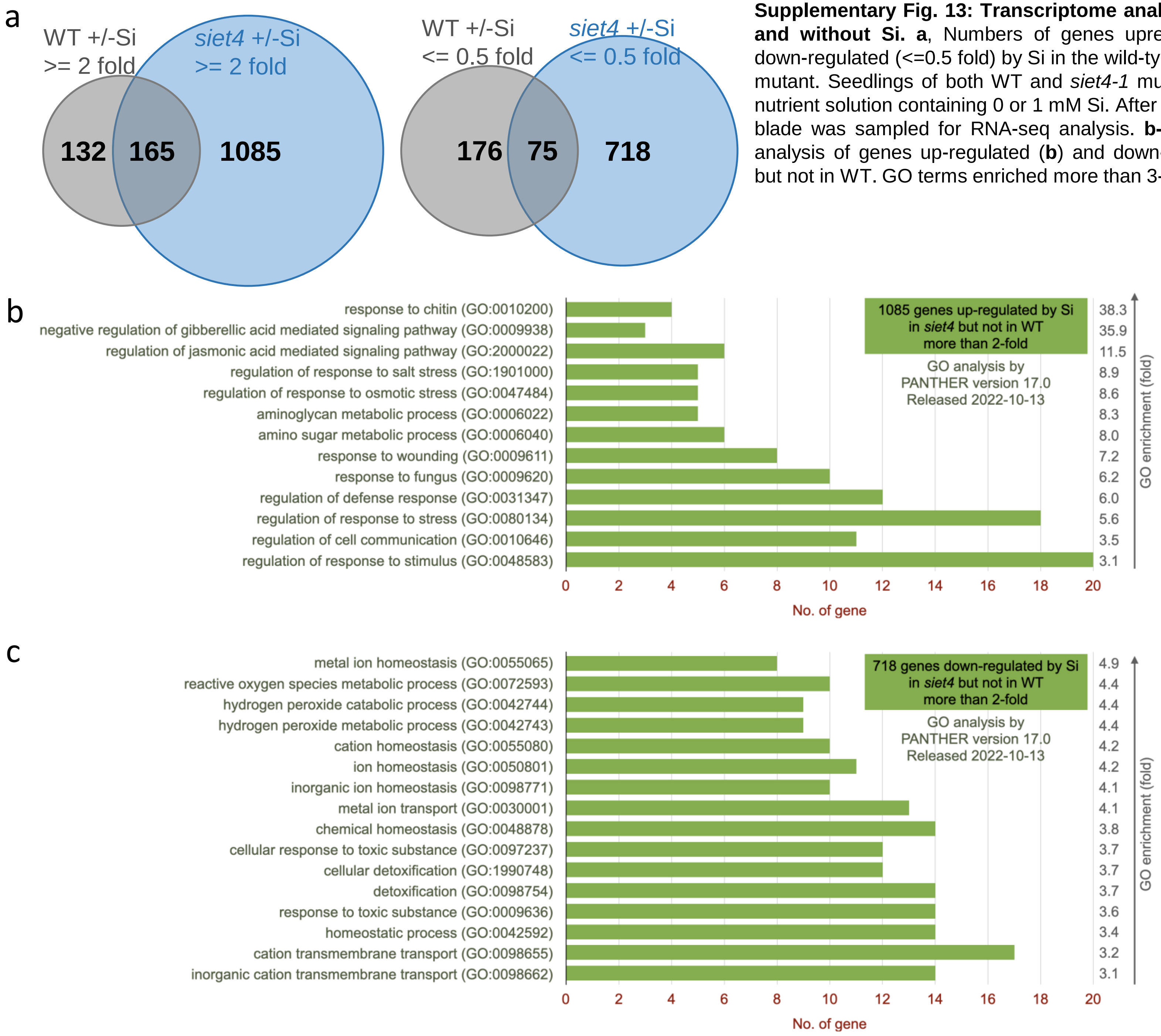

Supplementary Fig. 13: Transcriptome analysis of leaf blade with and without Si. a, Numbers of genes upregulated (>=2 fold) and down-regulated (<=0.5 fold) by Si in the wild-type rice (WT) and *siet4-1* mutant. Seedlings of both WT and *siet4-1* mutant were exposed to a nutrient solution containing 0 or 1 mM Si. After 1 day exposure, the leaf blade was sampled for RNA-seq analysis. **b-c**, Gene ontology (GO) analysis of genes up-regulated (**b**) and down-regulated (**c**) in *siet4-1* but not in WT. GO terms enriched more than 3-fold are shown.

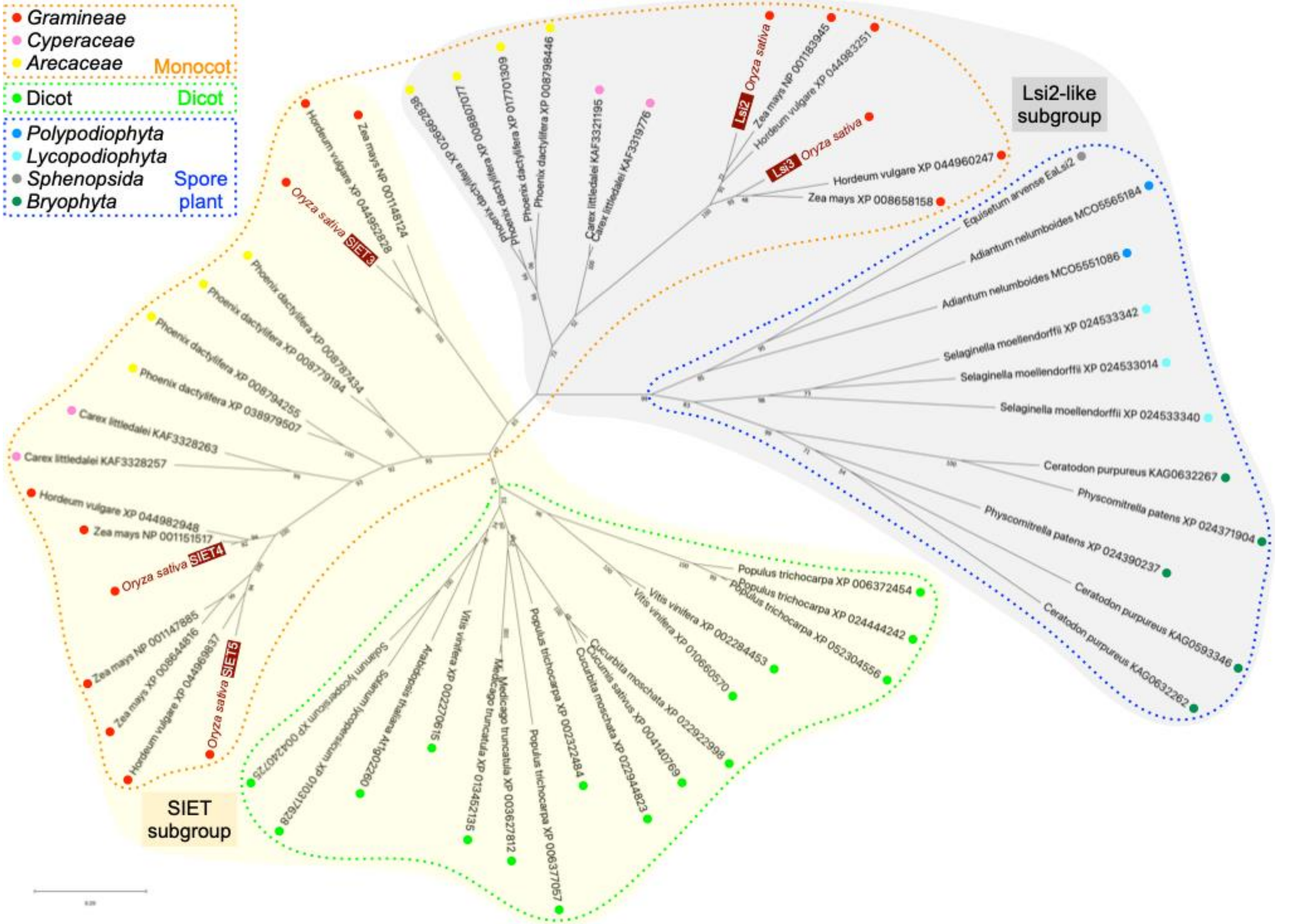

Supplementary Fig. 14. Phylogenetic tree of Lsi2/SIET-like proteins in plant kingdom. Phylogenetic tree was constructed by Maximum Likelihood method using MEGA X. Bootstrap values from 1,000 trials are indicated. The 0.2 scales show substitution distance. Two subgroups (Lsi2-like and SIET4-like) are shown in different background colors. Genes in different taxa are indicated with different colors.

Supplementary Table 1. Primer sequences used in this study.

Primer name	Forward (5'-3')	Reverse (5'-3')	Purpose
SIET4-crispr1	GTTGCAGGAGTGTGAATTCGGAGG	AAACCCTCCGAATTCACACTCCTG	CRISPR/Cas9 system
SIET4-crispr2	GTTGGGGATGTTTATTACGGTCGA	AAACTCGACCGTAATAAACATCCC	CRISPR/Cas9 system
SIET4-seq1	ATCAGGAGCAACAGCGTGAGGA	GCTGGCTAGCAGTTCTTGTG	Sequencing
SIET4-seq2	CTCAACTGTCACTGACAGGA	ATTGTTCTGAAGATTTACCTGTG	Sequencing
qPCR-SIET4	AAGCAGACGGTGATTGAGAAGG	GCATGTGCAGTTGTACAAACACC	qPCR
qPCR-Actin	GACTCTGGTGATGGTGTCAGC	GGCTGGAAGAGGACCTCAGG	qPCR internal control
qPCR-HistoneH3	GGTCAACTTGTTGATTCCCCTCT	AACCGCAAAATCCAAAGAACG	qPCR internal control
qPCR-Ubiquitin	AGAAGGAGTCCACCCTCCACC	GCATCCAGCACAGTAAAACACG	qPCR internal control
